# Neuroplasticity-driven technology-Assisted physiotherapy in cerebral dysfunction: A review of current evidence and clinical applications

**DOI:** 10.6026/973206300223534

**Published:** 2026-06-30

**Authors:** Nand Kishor Prasad Sah, Himani Rathi, Shipra Gangwar, Ranjit Tiwari, Simran Saxena, Pratibha Singh

**Affiliations:** 1 Department of Physiotherapy, Teerthanker Mahaveer University, Moradabad, India

**Keywords:** Neuroplasticity, cerebral dysfunction, technology-assisted rehabilitation, virtual reality, brain-computer interface

## Abstract

Long-term motor and functional disability following cerebral dysfunction remains a major global rehabilitation challenge despite advances in conventional physiotherapy approaches. Therefore, it is of interest to synthesise existing evidence on neuroplasticity and technology- based physiotherapy interventions for cerebral dysfunction and to evaluate their mechanistic foundations, clinical effectiveness and translational potential. Technology-based modalities such as robotic exoskeletons, VR systems, BCIs and non-invasive neuro-stimulation enhance cortical reorganisation, motor relearning and functional independence compared with traditional physiotherapy. Neuroplasticity- driven and technology-assisted physiotherapy represent a paradigm shift in the management of cerebral dysfunction, as evidenced by large- scale randomised controlled trials. Technology-assisted physiotherapy increases the neurorehabilitation outcomes regarding stroke, traumatic brain injury and cerebral palsy.

## Background:

Cerebral dysfunction is a broad term that includes stroke, traumatic brain injury (TBI) and cerebral palsy (CP), all of which are major causes of chronic disability and socioeconomic burden worldwide [1]. These disorders are characterized by structural and functional impairment of neural pathways that control motor, cognitive and sensory processes, leading to impairments that may be either focal limb weakness to complete functional dependency [2]. Physiotherapy is an established neurorehabilitation approach that supports motor recovery through task-specific movement practice, muscle-strengthening and functional retraining. However, conventional methods alone are often insufficient, particularly in individuals with severe or chronic neurological impairments, due to limited neurobiological engagement and plateauing of functional recovery [3]. Over the past two decades, there has been a paradigm shift in the understanding of the adult brain and its response to injury and therapeutic interventions. The neurobiological basis of recovery after brain injury is neuroplasticity, defined as the inherent ability of the brain to change its structure, function and connectivity based on experience. Importantly, neuroplasticity is not a passive process, but an activity- dependent experience-specific process that requires structured, high-intensity and meaningful motor practice to stimulate adaptive cortical reorganisation [4]. Advances in digital health technologies have led to the development of new rehabilitation tools. It includes robot-assisted therapeutic systems, immersive virtual reality (VR) environments, brain-computer interfaces (BCIs) and transcranial neurostimulation techniques that are uniquely placed to exploit and enhance neuroplastic mechanisms [5]. These technologies enable precise, repetitive and quantifiable motor training, provide real-time biofeedback and allow modulation of cortical excitability, thereby expanding the therapeutic potential of conventional physiotherapy [6]. Ischemic stroke remains a leading cause of long-term disability, and although neuroplasticity is fundamental to post-stroke recovery, rehabilitation outcomes depend on the balance between adaptive plasticity that restores functional neural networks and maladaptive plasticity that reinforces compensatory behaviors; therefore, this review examines physiotherapy strategies that optimize neuroplasticity- driven recovery, with particular emphasis on emerging biomarker-guided and multimodal rehabilitation technologies reported between 2024 and 2026 [7, 8]. Recent evidence highlights the substantial potential of neuroplasticity in stroke rehabilitation, while emphasizing that individualized responses, optimal intervention timing and duration, and sociocultural and clinical factors must be considered to maximize recovery outcomes through personalized and multidisciplinary rehabilitation strategies [9]. The objective of this narrative review is to synthesise existing evidence on the mechanistic basis of neuroplasticity in cerebral dysfunction and to determine the effectiveness of technology-based physiotherapy modalities. Therefore, it is of interest to report translational barriers and offer an integrative clinical model of developing rehabilitation practice in the constantly developing field.

## Neuroplasticity processes in cerebral dysfunction:

The multiple pathways of neuroplasticity after cerebral injury include synaptic potentiation, axonal sprouting, dendritic maturation and reorganization of cortical maps [4]. Adaptive remodeling of the perilesional cortex and contralesional hemisphere occurs during the acute and subacute phases of stroke, to compensate for damaged tissue. The cellular basis of motor memory consolidation and skill acquisition involves long-term potentiation (LTP) and long-term depression (LTD) of synaptic junctions [7]. The concept of Hebbian plasticity, which is also known as neurons that fire together wire together, provides the foundation to the principle that concomitant firing of pre- and post-synaptic neurons enhances the effectiveness of synaptic connections. This principle is applied therapeutically through synchronized volitional motor efforts with sensory feedback or external motor facilitation, such as BCI-based rehabilitation [5]. Diffuse axonal injury in TBI impairs large-scale network connectivity; however, pre-existing white matter tracts can be remodeled with the right therapeutic stimulation [3]. The developing brain in CP exhibits a higher degree of neuroplasticity; however, abnormal developmental patterns require early and sustained intervention to achieve functional cortical organization (Figure 1 - see PDF) [2].

## Robot-Assisted physiotherapy:

Robotic exoskeletons and end-effector devices are now established evidence-based therapeutic measures for upper and lower limb rehabilitation in stroke and TBI. Systems such as Lokomat, Armeo and ReWalk provide high-repetition, task-specific motor training with adjustable assistance levels, thereby reducing therapist workload and ensuring consistent movement quality [6]. A meta- analysis showed that robot-aided arm therapy results in a considerable change in motor activity (Fugl-Meyer scores) and activities of daily living than traditional therapy, especially when used in the subacute period [6].

Importantly, robotic devices promote neuroplasticity by enabling mass practice, a key driver of cortical map expansion and by providing proprioceptive and somatosensory feedback that reinforces motor learning loops. More recent models of soft robotic exosuits and electromyography (EMG)-controlled devices, include intention recognition, which provides active involvement of the patient, which is necessary in neuroplastic reorganization.

## Neurorehabilitation through virtual reality:

VR-based rehabilitation creates ecologically valid and immersive environments in which patients perform meaningful, goal-oriented motor tasks that stimulate motivational and attentional systems, thereby enhancing neuroplastic learning [8]. Randomized controlled trials (RCTs) demonstrate that VR is more effective than traditional therapy in improving upper limb function, gait and balance in survivors of chronic strokes [9]. VR interventions enhance bimanual coordination and upper limb movement quality in children by leveraging the enhanced cortical plasticity of the developing brain in CP. Accessible and cost- effective non-immersive VR systems (e.g., Nintendo Wii, Leap Motion) have demonstrated comparable short-term improvements in balance and walking speed among stroke patients. Gamification principles incorporated into VR platforms can improve exercise adherence, which is a vital in dose-response correlations of neuroplasticity-based rehabilitation.

## Brain-Computer Interfaces:

BCIs provide a direct communication pathway between the electrophysiological brain activity and external devices, enabling intentional motor output in patients with severe neuromotor impairment [5]. Based on electroencephalography (EEG)-recordings, BCIs detect motor imagery -related event-related desynchronization in the sensorimotor cortex and translate it into robotic movement or functional electrical stimulation (FES). Together with peripheral sensory input, this closed loop paradigm directly drives Hebbian plasticity loops with cortical motor commands. Clinical trials in chronic stroke have shown that BCI-controlled FES results in greater recovery of paretic hand function compared to FES alone, supported by neuroimaging evidence of improved ipsilesional corticospinal tract integrity [5]. BCI-based rehabilitation has emerged as a promising option for patients who do not respond to conventional physiotherapy, representing a new therapeutic frontier for individuals with chronic stroke (Table 1).

## Combination of non-invasive brain stimulation and physiotherapy:

Transcranial magnetic stimulation (TMS) and transcranial direct current stimulation (tDCS) can alter cortical excitability to produce favorable conditions to induce neuroplastic change [10]. Repetitive TMS applied over the ipsilesional motor cortex increases upper limb motor recovery by improving cortical excitability and when combined with physiotherapy yields significantly greater recovery than physiotherapy alone, as shown in several RCT and meta-analyses [10]. Another study reported that anodal tDCS enhanced synaptic plasticity in the ipsilesional motor cortex via NMDA receptor-mediated mechanisms, with additive effects when combined with task-specific training in stroke survivors. Transcranial stimulation studies in pediatric CP remain in early stages but are promising; with preliminary evidence indicating that combined cortical stimulation and physiotherapy improve selective motor control and gait parameters compared to sham stimulation [2].

## Current trends:

Recent advances in neurorehabilitation emphasize neuroplasticity-based interventions, including exercise therapy and non-invasive neuromodulation techniques such as transcranial magnetic stimulation, vagus nerve stimulation, and peripheral nerve stimulation, which have demonstrated potential to enhance motor recovery and functional restoration across neurological conditions while highlighting opportunities for personalized rehabilitation and combined therapeutic approaches [8]. The interplay between neuroplasticity science and rehabilitation technology has fundamentally redefined the limits of recovery following cerebral dysfunction. Current evidence supports the premise that technology-assisted modalities, including robotic systems, VR-based interventions, BCIs and neurostimulation, exert therapeutic effects by enhancing activity-dependent neuroplasticity rather than merely facilitating movement [4]. A key observation from the literature is the dose-response relationship between therapy intensity and neuroplastic reorganization. Technologies that enable high-repetition, high-intensity and goal-directed practice are associated with superior motor recovery outcomes, consistent with translational animal models that link training intensity to increased dendritic spine density and cortical map expansion [3]. Although there is strong evidence at the preliminary stage, there are still a lot of translational gaps. First, small sample sizes, heterogeneous outcome measures and short follow-up periods in most trials limit conclusive findings regarding long-term neuroplastic outcomes. Second, the optimal timing, dosage and sequencing of technology-assisted interventions at different stages of recovery remains poorly defined [1]. Third, high costs, infrastructure requirements and relatively small amount of clinician knowledge pose significant barriers to widespread clinical adoption, particularly in low- and middle-income countries such as India, where the burden of stroke and TBI is high [11]. Future research should focus on large-scale, multi-site RCTs with neuroimaging neuroplasticity biomarkers (diffusion tensor imaging, functional MRI), health economic analyses and the development of clinical decision-support systems to guide technology selection based on lesion characteristics, recovery stage and individual neuroplasticity profiles. Another opportunity is the use of artificial intelligence and machine learning algorithms in the rehabilitation robotics and BCI, to customize the training parameters in real time, representing a valuable avenue for future research (Table 2) [12].

## Conclusion:

The use of neuroplasticity and technology-aided physiotherapy is a scientifically based paradigm shift in cerebral dysfunction rehabilitation. Robotic exoskeletons, VR environments, BCIs and transcranial neuro-stimulation provide complementary mechanisms for enhancing amplifying cortical reorganization, motor relearning and functional recovery of stroke, TBI and CP. The development of these modalities into structured, high-intensity and patient-centered rehabilitation models has a substantial potential of overcoming the functional recovery limits that are realized with traditional physiotherapy.

## Advancement to Knowledge

This narrative review integrates current evidence on neuroplasticity-driven and technology-assisted physiotherapy modalities for cerebral dysfunction, including robotic rehabilitation, virtual reality, brain-computer interfaces and non-invasive brain stimulation. The review highlights the mechanistic relationship between neuroplasticity and functional recovery while addressing translational barriers, clinical applicability and future directions involving artificial intelligence-assisted rehabilitation. The study provides an integrative framework for modern neurorehabilitation practice in stroke, traumatic brain injury and cerebral palsy.

## Funding:

None

## Publication ethics statement:

The authors declare that this manuscript complies with COPE guidelines on publication ethics. The authors confirm that the work is original, has not been published elsewhere and is not under consideration by another journal. All authors approved the final manuscript and declare no unethical or misleading practices associated with this publication.

## Figures and Tables

**Table 1:** Overview of technology-assisted physiotherapy modalities and their mechanisms

**Modality**	**Mechanism of Action**	**Neuroplastic Effect**	**Clinical Applications**	**Key Advantages**
Robotic therapy	Repetitive task-specific training	Cortical map expansion	Stroke, TBI	High intensity, consistency
Virtual Reality	Immersive task-based learning	Sensorimotor integration	Stroke, CP	Motivation, engagement
BCI	EEG-based motor intent decoding	Hebbian plasticity	Chronic stroke	Direct brain activation
TMS/tDCS	Cortical excitability modulation	Synaptic plasticity	Stroke, CP	Non-invasive neuromodulation

**Table 2:** Translational challenges and future research directions in neuroplasticity-based rehabilitation

**Challenge**	**Description**	**Impact**	**Proposed Solution**
Small sample sizes	Limited statistical power	Weak evidence	Large multicenter RCTs
Heterogeneous outcomes	Variable endpoints	Poor comparability	Standardized metrics
High cost	Equipment/infrastructure	Limited access	Cost-effectiveness models
Limited expertise	Training gaps	Low adoption	Clinical training programs
Undefined protocols	Timing/dosage unclear	Suboptimal outcomes	Protocol optimization studies
